# Detection of Antimicrobial Resistance Genes Associated with Carbapenem Resistance from the Whole-Genome Sequence of *Acinetobacter baumannii* Isolates from Malaysia

**DOI:** 10.1155/2020/5021064

**Published:** 2020-04-02

**Authors:** Mohan Rao, Fairuz A. Rashid, Surianti Shukor, Rohaidah Hashim, Norazah Ahmad

**Affiliations:** ^1^Bacteriology Unit, Infectious Disease Research Centre, Institute for Medical Research, Ministry of Health, Kuala Lumpur, Malaysia; ^2^Infectious Disease Research Centre, National Institute of Health, Ministry of Health, Shah Alam, Malaysia

## Abstract

**Background:**

The spread of carbapenem-resistant *A. baumannii* (CrAb) is gaining worldwide attention. The spread of this pathogen is largely due to its ability to acquire various resistance genes of intrinsic and extrinsic origins that confer unpredictable susceptibility to *β*-lactams. The aim of this study was to analyze *β*-lactamase genetic compositions of CrAb in Malaysia.

**Methods:**

Whole-genome sequencing (WGS) was carried out on 13 CrAb isolates from clinical samples in Malaysia from 2011 to 2016.

**Results:**

Endotracheal aspirate was the dominant clinical sample source (*n* = 6), and only one isolate was obtained from wound swab. A total of 6 sequence types (STs) of the Oxford scheme were identified, including 4 reported STs and 2 novel STs. Eleven isolates were classified into clonal complex 92 (CC92/ICII), among which ST195 and ST208 were the most prevalent STs. All 13 CrAb isolates harbored multiple *β*-lactamase genes. *bla*_OXA-23_ (*n* = 13) and *bla*_OXA-66_ (*n* = 11) were the dominant carbapenemase gene families found in these isolates. All isolates harbor *bla*_ADC_, *bla*_OXA-51-like_, and *bla*_OXA-23-like_ genes. *bla*_TEM_ (*n* = 7), *bla*_NDM-1_ (*n* = 3), *bla*_CARB-8_ (*n* = 1), and *bla*_PER-3_ (*n* = 1) are amongst other *β*-lactamase genes found in this study. IS*Aba1* was found upstream to *bla*_OXA-23_ (*n* = 13), *bla*_OXA-66_ (*n* = 1), and *bla*_ADC_ (*n* = 11). All *bla*_NDM-1_ isolates had IS*Aba125* (mobile genetic element) upstream to the genes. All isolates were positive for Tn*2006/2008* and Tn*2009* but were negative for Tn*2007*.

**Conclusion:**

Most of the isolates were grouped under the CC92 clonal complex which belongs to international clonal lineage 2. These findings predict that carriage of carbapenem-resistant genes possibly constitutes the underlying basis of high level of international clone II prevalence. Therefore, molecular surveillance and antimicrobial stewardship are essential in implementing policies to prevent and control the spread of CrAb in hospital settings.

## 1. Introduction

*Acinetobacter baumannii* (*A. baumannii*) is an infectious agent that has been the leading cause of hospital-acquired infections [1]. It is an opportunistic pathogen that poses significant threat to public health and associated with high mortality [2]. *A. baumannii* nosocomial infection is now common throughout the world [3, 4]. Selection of an appropriate empirical antimicrobial agent is extremely difficult due to its unpredictable antimicrobial resistance genes which are commonly acquired via mobile genetic elements [5].

*A. baumannii* belongs to a group of clinically important organism, known as ESKAPE. It is predominantly found among health care-associated organisms that have the potential of substantial antibiotic resistance [6]. *A. baumannii* infection usually involves excretory organ systems that contain high level of fluids. The most common sites of infection are respiratory tract, urinary tract, and peritoneal cavity and highly associated with indwelling devices such as endotracheal tube, urinary catheter, Tenckhoff catheter, and intravascular catheter [7].

Carbapenem-resistant *A. baumannii* (CrAb) was identified as the critical organism based on the global priority pathogen list proposed by the World Health Organization (WHO). It has been concluded that development of new antimicrobial is the current focus globally [8]. CrAb has become a major concern among healthcare facilities due to its rising prevalence. In countries of the Arab League and Vietnam, prevalence of CrAb has been reported ranging from 50 to 88%, whereas in the United Kingdom, it ranges from 40 to 70% [9, 10]. According to the National Surveillance Antibiotic Resistance database, CrAb prevalence in Malaysia ranges from 50 to 60% and remained static since year 2008 up to 2016 [11]. However, several studies from different hospitals in Malaysia showed CrAb prevalence higher than the national surveillance [12].

Nonjudicious use of antibiotics has led *A. baumannii* to rapidly acquire antimicrobial resistance genes from the environment. At the same time, selective antimicrobial pressure induces genome rearrangement associated with chromosomally (intrinsic) encoded antimicrobial resistance genes which has resulted in transposition of insertion sequence (IS) as a promoter of various CHDLs [13]. *A. baumannii* possesses *bla*_OXA-51-like,_ an intrinsic carbapenem-hydrolysing oxacillinase gene. The expression of this gene may vary with the presence of IS*Aba1* as a promoter [14]. It also acquires certain *bla*_OXA_ and *bla*_non-OXA_ group genes from plasmids [15]. Predominantly acquired *bla*_OXA-group_ gene is *bla*_OXA-23-like_, whereas the most prevalent *bla*_non-OXA_ group gene is *bla*_NDM-1_ [16].

This study is aimed to analyze the molecular characteristics of 13 *A. baumannii* isolates obtained from hospitalized patients in Malaysia with underlying carbapenem-resistant phenotype.

## 2. Methods

Of 1933 *A. baumannii* isolates collected from various hospitals throughout Malaysia from year 2011 to 2016, we selected 13 carbapenem-resistant *A. baumannii* (CrAb) isolates that were resistant to carbapenems. No genes (*bla*_NDM_, *bla*_OXA_, *bla*_KPC_, *bla*_VIM_, and *bla*_IMP_) were found using in-house PCR. These isolates were recovered from patients receiving intensive care and were isolated from respiratory secretion, urine, rectal swabs, and pus. The initial identification test based on biochemical methods was performed using API 20E (bioMérieux, LaPlane, France). Antimicrobial susceptibility was determined by the disc diffusion method for gentamicin, amikacin, ciprofloxacin, cefepime, ceftazidime, aztreonam, imipenem, meropenem, ertapenem, and ampicillin-sulbactam according to Clinical and Laboratory Standards Institute (CLSI) criteria. Minimum inhibitory concentrations (MICs) of imipenem, meropenem, and ertapenem were determined by the E-Test method according to CLSI criteria. CrAb is defined as an *A. baumannii* isolate that is resistant to meropenem, ertapenem, and imipenem with an MIC value of ≥4 *μ*g/ml via ETest.  Table 1 summarizes study isolates, types of specimen, and the genebank identification.

Total DNA of these strains was extracted by using a MasterPure™ DNA Purification Kit (Epicentre, Madison, Wisconsin, USA) and quantified using Qubit 2.0® Fluorometer (Life Technologies, Carlsbad, CA). DNA libraries were prepared using a Nextera DNA Flex Library Prep Kit (Illumina Inc.), according to the manufacturer's instructions. Sequence data for all strains were obtained using an Illumina Nextseq platform (Illumina Inc., San Diego, CA, USA). Raw sequence quality trimming was carried out as described by SPAdes version 3.9.1 for de novo assembly and confirmation [17].

Average nucleotide identity (ANI) was calculated by using a gANI tool calculator, ANI calculator software version 1.0. ANI values above 95% between genomes of these isolates denote the same species [18]. Multilocus sequence typing (MLST) analysis was streamlined via the MLST program against PubMLST database via MLST version 2.6 software. Oxford scheme of *A. baumannii* was used for MLST analysis. The 7 housekeeping genes were *gltA*, *gyrB*, *gdhB*, *recA*, *cpn60*, *gpi*, and *rpoD* [19]. New alleles and STs were submitted to the curator of the database, and new ST numbers were allotted. Clonal complexes were assigned by eBURST and were defined as single locus and double-locus variants with an outgroup *A. baumannii* strain ATCC 17978 as a reference (GenBank accession number: CP000521.1) [20]. Antimicrobial resistance genes (AMR) were confirmed by the CARD and resistance gene identifier (Resfinder) via Abricate-Version 0.8 software [21]. kSNP version 3.0 was used to identify pan-genome single-nucleotide polymorphism (SNP) [22]. A SNP phylogenetic tree was drawn based on pairwise whole-genome sequence via Type Genome Server using multiple reference strains that belong to the *Acinetobacter baumannii* complex group. Ugene-PRO and ISfinder applications were used to analyze the presence of mobile genetic elements [23]. The sequence of this whole-genome shotgun project has been deposited in GenBank under Genome submission: SUB5536145 with the BioProject ID: PRJNA539835.

## 3. Results

The genome sequence size of the 13 isolates in this study ranged from 3,8321,210 to 4,246,682 base pair (bp), with contigs ranging from 70 to 104, which encodes 3577 to 4003 coding sequences, 50 to 53 tRNA, 3 rRNA, and 1 tmRNA. Six of 13 (46%) isolates were obtained from endotracheal aspirate followed by urine culture (4 (31%)), rectal swab (2 (15.4%)), and wound swab (1 (7.7%)). No isolate of CrAb was cultured from blood samples. Average nucleotide identity (ANI) of all isolates was above 95% which concludes that they belonged to the same species of bacteria. However, 3 paired isolates shared 100% identity of ANI although obtained from different sources and states. Those isolates were CRE400/16-CRE245/15, CRE645/16-CRE648/15, and CRE1071/16-CRE1159/16. Isolates from our study were compared with a genome of reference strain. The SNP-based phylogenetic tree showed that most of the isolate genomes were closely associated with each other and belonged to international clone II.

**Figure 1:**
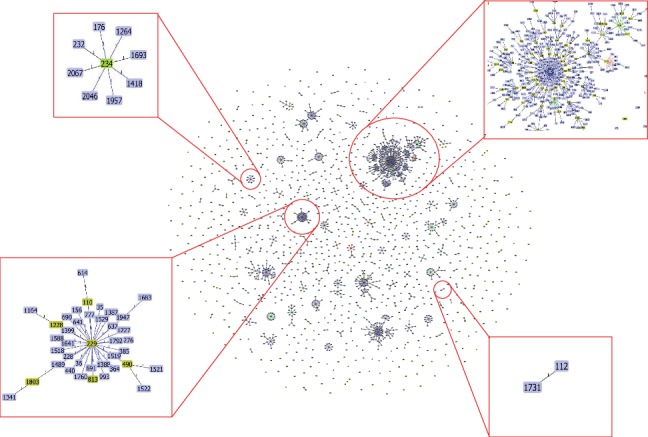
Population snapshot of *A. baumannii*. Clusters of related STs and individual unlinked STs within the entire *A*. *baumannii* Oxford MLST database are displayed as a single eBURST diagram by setting the group definition to zero of seven shared alleles. Clusters of linked isolates correspond to clonal complexes. STs found in this current study are magnified in the box beside. *A. baumannii* strain ATCC 17978 (reference) belongs to ST112.

MLST analysis with the Oxford scheme in this study revealed a total of 4 defined STs and 2 novel STs (Figure 1). The 2 novel STs of year 2014-2015 were submitted and were assigned as ST1947 and ST1948. ST195, accounting for the largest proportion (5/13, 38%), was the major clonal type followed by ST208 (3/13, 23%), ST938 (2/13, 15.4), ST1418 (1/13, 7.7%), ST1947 (1/13, 7.7%), and ST1948 (1/13, 7.7%). Additionally, ST195, ST208, ST938, and ST1948 were double-locus variants of *gyrB*, *gbhB*, and *gpi* genes interchangeably. eBURST analysis showed that these 3 defined STs along with one novel ST clustered in the same CCs (CC92), which was also referred to as global clonal 2 (GC2)/international clonal II (ICII).

All isolates harbored intrinsic *bla*_OXA-51-like_ class D carbapenemases. *bla*_OXA-66_ was the most prevalent 11 (85%), followed by *bla*_OXA-64_ and *bla*_OXA-91_, 1 (8%) of each, respectively. An extrinsic *bla*_OXA_-type carbapenemase gene found was *bla*_OXA-23_ (100%), while no isolates contained *bla*_OXA-24-like_ or *bla*_OXA-58-like_ gene. Interestingly, all isolates harbored more than one oxacillinase gene. Acinetobacter-derived single-variant cephalosporinase *bla*_ADC_ gene was present in all our isolates. A total of 8 (62.5%) isolates harbored class A *β*-lactamase gene *bla*_TEM_, 1 isolate carried *bla*_CARB_, and 1 isolate carried *bla*_PER_ gene. In addition, 3 (23.1%) isolates carried class B metallo-*β*-lactamase (MBL) gene *bla*_NDM_. Additionally, all isolates were negative for other MBL genes which included *bla*_IMP_, *bla*_VIM_, *bla*_GIM_, and *bla*_SPM_.  Table 2 summarizes all the AMR genes detected in this study.

As described in [24], we found that 1 (7.7%) isolate harbors class 1 integron. The presence of mobile genetic element provides strong evidence for the horizontal dissemination of antibiotic resistance genes. At the same time, all *bla*_OXA-23-like_ genes were carried by Tn*2006* and Tn*2008* in our study. In addition, we detect insertion sequence (IS) elements in the promoter regions of several AMR genes [25]. All isolates with *bla*_NDM_ gene were found to harbor IS*Aba125* upstream to the corresponding gene *bla*_NDM_. IS*Aba1* was found upstream to *bla*_OXA-23_ (*n* = 13), *bla*_ADC_ (*n* = 11), *bla*_OXA-66_ (*n* = 1), and *bla*_CARB_ (*n* = 1). IS*91* was found upstream to *bla*_PER_ (*n* = 1).

## 4. Discussion

*A. baumannii* of nosocomial origin has been the leading cause of hospital-acquired infections [1]. The nature of this bacterium is that it can be found in the environment, intrinsically carrying the antibiotic resistance gene and posing a significant threat to public health due to its unpredictable antibiotic susceptibility [2, 4, 5]. This study was aimed to determine the genetic mechanisms conferring carbapenem resistance in our local strains.

Most of the clinical isolates in this study obtained from respiratory secretion (tracheal aspirate, sputum, and bronchial alveolar lavage) were similar to the Malaysian local surveillance study. Up to year 2017, nosocomial *A. baumannii* was commonly isolated from respiratory secretion, followed by blood isolation [11, 26]. Likewise, many studies nationwide shared similar findings. *A. baumannii* preferably colonizes or infects the respiratory tract. Such infection commonly occurs in debilitated patients especially in the ICU. Patients of mechanical ventilation and lengthy hospital stay are at risk of *A. baumannii* infection [27, 28].

**Figure 2:**
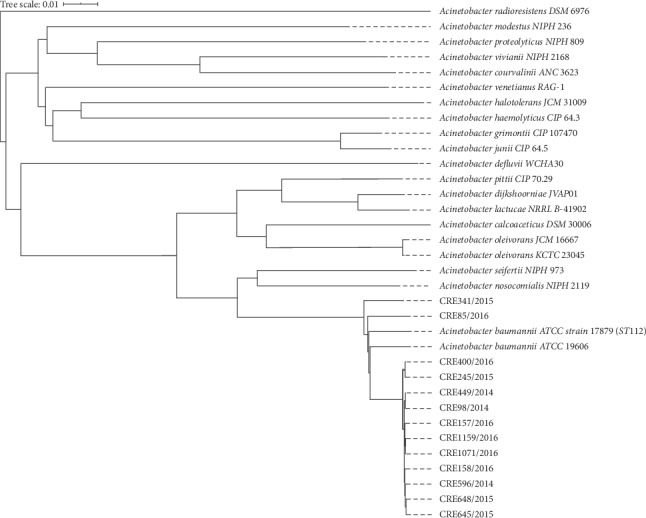
Phylogenetic tree of single nucleotide polymorphisms from whole-genome sequencing was drawn based on pairwise comparison via Type Genome Server. It includes reference strains of other *Acinetobacter baumannii* complex.

**Figure 3:**
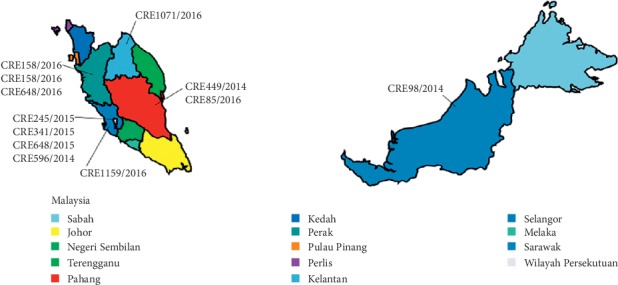
Geographic distribution of carbapenem-resistant *A. baumannii* strains according to different states in Malaysia from year 2011–2016.

ST195 was the frequent sequence types observed in our study. At the same time, as expected, most of these CrAb isolates belong to the CC92/IC2 clonal lineage. The predominance of CC92/IC2 in the present study was similar to reports produced in other neighbouring Asian countries such as China and Thailand and consistent with local studies and reports [29–31]. We also found novel strains of different clonal lineage emerging. Based on our study, these new strains have emerged in 2014 and 2015. In [19, 28, 32], the authors have revealed that emergence of newer strains is caused by inappropriate antibiotic usage. It is crucial to study about epidemiology of sequence types as there is positive correlation between clonal complex and *bla*OXA carriage. Single-locus sequence-based typing of *bla*_OXA-51-like_ genes assigns 11 clinical samples of this study to a single clonal complex [33]. CC92 clonal lineage isolates commonly harbor *bla*_OXA-23_ and *bla*_OXA-66_, similar to the findings in this study [34]. Figures 1–3 portray the SNP phylogenetic tree, minimum spanning tree (MST), and the distribution of the studied isolates in Malaysia.

In clinical microbiology laboratories, *A. baumannii* is indistinguishable with other species of the *Acinetobacter calcoaceticus-Acinetobacter baumannii* complex by widely used routine identification systems due to its similar phenotypic and biochemical properties. The accurate identification of *A. baumannii* is only possible via molecular methods. Molecular characterization of *bla*_OXA-51-like_ gene detection is carried out along with RNA polymerase *β*-subunit gene (*rpoB*) and DNA gyrase B gene (*gyrB*) for *A. baumannii* species identification [35]. All the isolates involved in this study were positive for *bla*_OXA-51-like_, *rpo*B, and *gyr*B genes. This finding resonates along with SNP phylogeny and ANI on species-level identification in this study.

*A. baumannii* is known for its enzymatic degradation mechanism by *β*-lactamases [36]. The most common carbapenem resistance mechanism found in our study was the existence of various *β*-lactamases and mobile genetic elements. *bla*_OXA-23-like_ and *bla*_OXA-51-like_ were the most prevalent, accounting for 100% carbapenem resistance amongst studied isolates. *bla*_OXA-51-like_ gene was detected in all the isolates due to its chromosomal-borne nature, naturally occurring in oxacillinase gene [37].

Meanwhile, *bla*_OXA-23-like_ gene can be either plasmid or chromosome-borne, resulting in increased rates of carbapenem resistance in healthcare settings due to its mobility in facilitating horizontal genetic transfer. The acquisition of *bla*_OXA-23-like_ gene is a major public health concern for its horizontal dissemination and rapid spread [38].

No isolates harbored *bla*_OXA-24-like_, *bla*_OXA-48-like_, and *bla*_OXA-58-like_ genes. Although these genes are disseminated in Europe and Middle East, they remained rare in our local findings [39, 40]. A variant of *bla*_OXA-51-like_ found in this study, namely, *bla*_OXA-66_ is commonly found in China [41]. At the same time, the present study observed that both *bla*_OXA-23-like_ and *bla*_OXA-51-like_ genes are found in all the isolates. These findings were similar to [42] as it common to find *A. baumannii* isolates harbor *bla*_OXA-23-like_ and *bla*_OXA-51-like_ in the Asian continent, whereas *bla*_OXA-51-like_ and *bla*_OXA-58-like_ in the western hemisphere.

MBL carrying *A. baumannii* isolates are rare nationwide. *bla*_NDM_ carrying *A. baumannii* is not commonly found in our area of study. We have not encountered *bla*_NDM_ during previous years or among many local studies [38, 39]. However, to our surprise, a small number (3/13, 24%) of *A. baumannii* isolates collected during 2014–2016 were positive for *bla*_NDM_, indicating recent emergence. Worthy of mentioning is the fact that the isolates carrying *bla*_NDM_ gene did not belong to the same clone lineage [43]. This will be the first reporting on *bla*_NDM_ gene harboring *A. baumannii* isolates from clinical samples in Malaysia.

Similar to the *bla*_OXA-51-like_ oxacillinase gene, *bla*_ADC_ is also a chromosomally encoded acinetobacter-derived cephalosporinase gene found among all the *A. baumannii* isolates in this study. This finding indicates intrinsic-species specific gene [44]. *bla*_TEM_ genes were also found in ¾ of our isolates. In [45], the authors had demonstrated that carbepenem resistance among CrAb is due to the coexistence of *bla*_OXA-23_ and *bla*_TEM_. Similar output was observed in this study; however, isolates without *bla*_TEM_ also exhibit the resistant phenotypes. To a moderate level, *bla*_CARB_ and *bla*_PER_ were also detected. No prevalence of colistin-resistant genes was found among these isolates. However, there are *A. baumannii* isolates found in many case reports from neighbouring countries such as Taiwan, India, and China that are resistant and becoming resistant [46].

CRE341/15 isolate harbors integrase class 1 indicating sporadic clones. Isolates carrying mobile elements such as integron-encoded integrase gene flanking resistance genes are capable of acquiring and transferring virulence genes via recombination [47]. Transposon played a major role in dissemination of resistant genes. In this study, we observed the presence of transposon Tn*2006/2008* in all the isolates carrying *bla*_OXA-23_ gene. This finding suggests that *bla*_OXA-23_ dissemination might be due to transposition of transposon [48]. In [14], the authors demonstrated that insertion of IS elements upstream to the resistant genes changes the expression level leading to the increased antimicrobial resistance phenotype. Every isolates in this study was found to have IS*Aba*1 upstream to *bla*_OXA-23_, *bla*_OXA-66_, *bla*_ADC-7_, and *bla*_CARB_. The IS*Aba125* was also found in the promoter region of all *bla*_NDM_ positive isolates. These findings suggest that the isolates may have other additional mechanisms resistance against carbapenem [49–51].

In summary, we demonstrated the possible clones of *A. baumannii* resistant to carbapenem and the prevalence of antibiotic resistance genes associated with mobile genetic elements. These findings provide epidemiological data of prevalent local STs as they are getting more diverse and resistant to multiple antibiotics. The presence of insertion sequence may reflect that these organisms readily take up external DNA. These findings are worrisome for its capability of outbreaks and horizontal resistance gene transmission. Molecular surveillance and antimicrobial stewardship are essential in implementing policies to prevent and control the spread of CrAb in hospital settings.

## Figures and Tables

**Table 1:** Study isolates, specimen types, biosample no., and GenBank accession number for *A. baumannii* isolates.

Isolate	Specimen type	NCBI biosample no.	GenBank accession no.
*A. baumannii* CRE1071/16	Pus	SAMN11513371	SWLT00000000
*A. baumannii* CRE1159/16	Urine	SAMN11513372	SWLS00000000
*A. baumannii* CRE157/16	Urine	SAMN11513373	SWLR00000000
*A. baumannii* CRE158/16	Endotracheal aspirate	SAMN11513374	SWLQ00000000
*A. baumannii* CRE245/15	Rectal swab	SAMN11513375	SWLP00000000
*A. baumannii* CRE341/15	Urine	SAMN11513376	SWLO00000000
*A. baumannii* CRE400/16	Endotracheal aspirate	SAMN11513377	SWLN00000000
*A. baumannii* CRE449/14	Endotracheal aspirate	SAMN11513378	SWLM00000000
*A. baumannii* CRE596/14	Endotracheal aspirate	SAMN11513379	SWLL00000000
*A. baumannii* CRE645/15	Rectal swab	SAMN11513380	SWLK00000000
*A. baumannii* CRE648/15	Endotracheal aspirate	SAMN11513381	SWLJ000000000
*A. baumannii* CRE85/16	Urine	SAMN11513382	SWLI000000000
*A. baumannii* CRE98/14	Endotracheal aspirate	SAMN11513383	SWLH00000000

**Table 2:** Resistance genes present in carbapenem-resistant *A. baumannii* from year 2011–2016.

Strain ID	State	Source	Oxford MLST	Clonal assigned	blaTEM	blaPER	blaOXA-23	blaOXA-51-like			blaNDM	blaADC	blaCARB	Mobile genetic elements				Transposons			
								blaOXA-66	blaOXA-91	blaOXA-64				ISAba1-ADC	ISAba1‐OXA23‐like	ISAba1‐OXA51‐like	ISAba125-NDM	Tn2006	Tn2007	Tn2008	Tn2009
CRE1071/16	Kelantan	Pus	ST195	CC92/IC2	−	−	+	+	−	−	−	+	−	+	+	+	−	+	−	+	−
CRE1159/16	Selangor	Urine	ST195	CC92/IC2	−	−	+	+	−	−	−	+	−	+	+	+	−	+	−	+	−
CRE157/16	Perak	Urine	ST195	CC92/IC2	−	−	+	+	−	−	−	+	−	+	+	−	−	+	−	+	−
CRE158/16	Perak	ETT	ST195	CC92/IC2	+	−	+	+	−	−	−	+	−	+	+	−	−	+	−	+	−
CRE245/15	Kuala Lumpur	Rectal Swab	ST938	CC92/IC2	−	−	+	+	−	−	−	+	−	+	+	+	−	+	−	+	−
CRE341/15	Kuala Lumpur	Urine	ST1947	CC229	−	+	+	−	−	+	+	+	−	−	+	−	+	+	−	+	−
CRE400/16	Perak	ETT	ST938	CC92/IC2	+	−	+	+	−	−	−	+	−	+	+	+	−	+	−	+	−
CRE449/14	Pahang	ETT	ST1948	CC92/IC2	+	−	+	+	−	−	+	+	−	+	+	+	+	+	−	+	−
CRE596/14	Kuala Lumpur	ETT	ST208	CC92/IC2	+	−	+	+	−	−	−	+	−	+	+	+	−	+	−	+	−
CRE645/15	Perak	Rectal Swab	ST208	CC92/IC2	+	−	+	+	−	−	−	+	−	+	+	+	−	+	−	+	−
CRE648/15	Kuala Lumpur	ETT	ST208	CC92/IC2	+	−	+	+	−	−	−	+	−	+	+	+	−	+	−	+	−
CRE85/16	Pahang	Urine	ST1418	CC234	−	−	+	−	+	−	+	+	+	−	+	+	+	+	−	+	−
CRE98/14	Sarawak	ETT	ST195	CC92/IC2	+	−	+	+	−	−	−	+	−	+	+	+	−	+	−	+	−

+indicates the presence of the resistant gene. MLST sequence type (ST) along with clonal complex is included in the table. *bla*_OXA_, *bla*_NDM_, *bla*_ADC_, *bla*_TEM_, and *bla*_PER_ are the *β*-lactamase genes identified. IS: mobile genetic element-insertion sequence; Tn: transposon; ETT: endotracheal tube.

## Data Availability

Genome database of this study is available in National Center of Biotechnology Information, NCBI.

## Additional Points

*Highlights. *(i) The spread of carbapenem-resistant *Acinetobacter baumannii* is gaining global attention. (ii) It is an opportunistic pathogen that poses a significant threat to public health and is associated with high mortality. (iii) Selection of an appropriate empirical antimicrobial agent is extremely difficult due to its unpredictable susceptibility patterns. (iv) The association of a mobile genetic element with the resistant gene is worrisome and presents as an emerging threat to our healthcare settings.
